# Belonging: a meta-theme analysis of women’s community-making in group antenatal and postnatal care

**DOI:** 10.3389/fpubh.2025.1506956

**Published:** 2025-02-26

**Authors:** Anna Horn, Marsha Orgill, Deborah L. Billings, Wiedaad Slemming, Astrid Van Damme, Mathilde Crone, Malibongwe Gwele, Nathalie Leister, Ashna D. Hindori-Mohangoo, Katrien Beeckman, Susan Bradley, Manodj P. Hindori, Jedidia Abanga, Julia Ryan, Hana Bucinca, Christine McCourt

**Affiliations:** ^1^Centre for Maternal and Child Health, City St. George’s, University of London, London, United Kingdom; ^2^Children’s Institute, Department of Paediatrics and Child Health, Faculty of Health Sciences, University of Cape Town, Cape Town, South Africa; ^3^Group Care Global, Philadelphia, PA, United States; ^4^University of South Carolina, Columbia, SC, United States; ^5^Vrije Universiteit Brussel, Brussels, Belgium; ^6^Department of Health Promotion, Maastricht University, Maastricht, Netherlands; ^7^University of Cape Town, Cape Town, South Africa; ^8^University of São Paulo, São Paulo, Brazil; ^9^Foundation for Perinatal Interventions and Research in Suriname (Perisur), Paramaribo, Suriname; ^10^Presbyterian Church of Ghana Health Service, Accra, Ghana; ^11^University of California, Berkeley, Berkeley, CA, United States; ^12^Action for Mothers and Children, Prishtina, Kosovo, Albania

**Keywords:** group care, maternity care, meta-theme analysis, belonging, health inequities

## Abstract

Health care systems are social institutions simulating microcosms of wider societies where unequal distribution of power and resources translate into inequities in health outcomes, experiences and access to services. Growing research on participatory women’s groups positively highlights the influence of group-based care on health and wellbeing for women, their infants, families and wider communities across different countries. With similarities in ethos and philosophies, group care combines relational, group-based facilitation and clinical care, uniquely offering an opportunity to examine the intersections of health and social care. With collated data from Group Care for the First 1000 Days (GC_1000), we conducted a qualitative meta-thematic analysis of women’s experiences of group antenatal and postnatal care in Belgium, Ghana, Kosovo, The Netherlands, South Africa, Suriname and The United Kingdom to better understand how and to what extent community-making engenders a sense of belonging amongst group care participants and how these experiences may address social well-being and health. Results from this analysis expose that women actively participate in community building in group care in three key ways: (1) Collective agreements, (2) Boundary setting and (3) Care Gestures, orchestrated via socio-spatial building embedded in key pillars of the model. This analysis also illustrates how a sense of belonging derived from group care can mobilise women to support and care for the wider community through communal building of health literacy which builds from individual to communal empowerment: (1) Individual Health, (2) Community Health, (3) Partner Involvement, (4) Social Care and (5) Including Wider Community in Group Care. This research study builds upon existing evidence from both group care and participatory women’s group literature, showcasing the potential of group-based care to holistically address women’s needs. This research further illustrates the ways women create a sense of belonging in the context of group care and highlights why belonging may be an integral component of the model’s facilitation of improved health and well-being for individuals as well as their wider communities. More research is needed to understand the link between belonging and community mobilisation in the context of group care and how it may address the needs of underserved communities.

## Introduction

Health care systems are a microcosm of society, as they are a core social institution that reflects the unequal distribution of power and resources observed in the wider world and the social context of individuals lives (1). Though contextual factors vary, sectors of the population in many countries remain underserved by maternity care services, leaving women, children, and their families at risk for poorer health outcomes during pregnancy, birth and the early postnatal period compared to more politically and socioeconomically privileged groups (2). Addressing infrastructural challenges, health care worker shortages and maternal–infant clinical outcomes remain high on the agenda for many public health initiatives whilst simultaneously, growing attention is placed on the relational dimensions of care that have the power to enhance (e.g., respectful maternity care, humanising birth) or inhibit (e.g., obstetric violence, obstetric racism, disrespectful care) positive maternal–infant health outcomes and experiences of providing and receiving services. Various factors influence a woman’s access to maternity care as well as the possibility of experiencing services that meet her needs holistically. Relationship-centred maternity care addresses inequities through continuity of support and engagement with the social factors that, in part, drive health and access to services.

Broadly defined, women’s groups refer to organised gatherings of women who come together to socialise, to support one another and/or to advocate for shared principles (3). We use women’s groups here as an umbrella term to describe group models of health and social care used to improve maternal and infant health and well-being. Research on women’s groups illustrates how positive relationships, including but also extending beyond the patient-clinician dyads, enhance health and social measures of wellness. For example, women’s groups may draw upon participatory and action learning theory which place group participants at the helm of decision making and problem solving regarding their health as opposed to being passive recipients of public health messaging. Evidence from participatory women’s group studies indicate they can address direct and indirect determinants of maternal and infant health, including socioeconomic factors (4–6). The Womb Trial, a mixed-methods study, developed a protocol to assess and measure the effectiveness of participatory women’s groups amongst Aboriginal and Torres Straight Islanders to improve maternal and child health (7). Other studies have also contributed to growing evidence in support of women’s groups, with an aim to understand the effectiveness of women’s participatory groups and key contextual factors for successful implementation – Tripathy et al. (8) studied groups in rural Eastern India; Sikorski et al. (9) investigated postnatal groups in high income countries; Houweling et al. (10) found that women’s groups improve infant mortality in low resource settings; and Prost et al. (11) reviewed cost effectiveness and improved chances of maternal and infant mortality in low resource settings.

Group antenatal and postnatal care (GANC and GPNC) also draws upon the benefits of women working together in group settings, amalgamating this approach with clinical health care. The overarching components are group facilitation, interactive learning, clinical checks with a maternity provider and self-checks (e.g., blood pressure readings, urine samples, weight) and community building (12). The distinction between group care and participatory women’s groups is the facilitation by at least one maternity care provider (most often a midwife) and the component of clinical care packaged within the group space, and in common with their shared ethos of participatory learning, women actively engage with their clinical care through the self-checks and interactive discussions, with professionals providing a more facilitative rather than didactic role. The model consists of a group of 6–12 pregnant women of similar gestational age (when possible) who regularly meet with two facilitators (at least one of whom is a clinician) in a group setting, preferably in a community location. The group continues to meet during one or more visits during the postnatal period. Continuity of both group participants and facilitators is a key pillar of the model design (13). Involvement of birth supporters (e.g., fathers, husbands, partners, birth companions) may be decided at the initial meeting of group care. Evidence to include partners, particularly male partners is mixed (14), however some cohorts decide to involve partners throughout antenatal and postnatal group care (15).

Whereas conventional antenatal and postnatal care tend to be reactive to health’s social influences, addressing mostly downstream results, health care workers in the group care model are instead trained as group facilitators, with skills in listening and involving the broader concerns of participants in interactive information exchange. Through the model, standard practices in public health messaging and perinatal care are redesigned - challenging, and sometimes disrupting top-down approaches to address the gaps in the reach of maternity services through knowledge exchange and community building (16). The egalitarian structure of the model draws upon knowledge diversity, positioning service users (group participants) as well as service providers (group facilitators) as knowledge holders with interconnected skills and information worth sharing and key to mutual learning (17). In this way, the model moves away from the deficit narrative presented in public health messaging, which many times alienates underserved groups through labels such as ‘vulnerable’ and/or ‘hard-to-reach.’ Many group health care models in women’s reproductive and maternal health share an ethos, design and delivery method to buttress the effects of belonging, particularly in comparison to conventional one-to-one care and uniquely places them as an avenue for communal impact and change.

The Group Care for the First 1000 Days (GC_1000) project was initiated in 2020 across seven different countries: Belgium, Ghana, Kosovo, The Netherlands, South Africa, Suriname and the United Kingdom to understand influences on implementation, sustainability and upscaling of GANC and GPNC, any contextual adaptations needed and the experiences of facilitators and pregnant and postnatal participants across diverse settings (18). Branching from CenteringPregnancy (and other variants of group care in the United States: BlackCentering, BELovedBIRTH, Melanated Group Midwifery Care), group care research outside of the United States has established itself in scientific literature for nearly a decade in countries in both the Global North (19, 20) and the Global South (6, 21, 22). Many of the GC_1000 research settings, especially Belgium, the Netherlands and the UK, centre on utilising the group model to address the needs, experiences and health inequities amongst women most underserved in maternity care (e.g., ethnic minority groups, refugees, non-speakers of the dominant language of the respective countries). Group care is gaining recognition for its positive impact in various settings (23) and the World Health Organization has named GANC a promising practice to be implemented in the context of research (24). Diverse research methodologies have been applied to understand the model, from ethnography (25) to randomized control trials in the Research for Equitable Antenatal Care and Health (REACH) Pregnancy Programme (20) and implementation science research in the multi-national GC_1000 project (18).

In group care research, women report increased satisfaction with the model of antenatal care compared to conventional one-to-one care, with some evidence indicating that group care may offer a route to culturally sensitive care (17, 26). Other studies also highlight the positive value women placed on the benefits of the group’s connectivity (27), enabling them to build community (28) and develop deeper support networks (29). In this article, we further explore these threads of connection and community building exhibited in the literature (30) and go deeper into the concept of belonging that women experience within the group. We centre the cultivation of ‘a sense of belonging’ to enhance not only experiences and outcomes of services, but also to build upon women’s relationship to health and well-being outside of the parameters of pregnancy and birth. We also consider how ‘a sense of belonging’ shapes women’s relationships with their own health, well-being, their local health services and the communities in which they live.

The concept of ‘belonging’ is polysemous, with a range of definitions from those of human geographers to anthropologists and philosophers. We draw on Marco Antonsich’s analytical framework, which explores belonging as “a discursive resource which constructs, claims, justifies, or resists forms of socio-spatial inclusion/exclusion (politics of belonging)” and then settle specifically on Probyn’s iteration in Antonsich’s argument which theorises belonging as “a mode of affective community-making based on physical proximity rather than a common identity” (31). Unlike Probyn’s exclusion of common identity, we instead situate ‘belonging’ precisely as “a mode of affective community-making” in the context of a relational form of antenatal and postnatal group care where shared identity amongst diverse group members is found in pregnancy, parenting and social identities. In an analytical framework for belonging, Antonsich outlines how identity is often entangled in definitions of belonging in the form of race, ethnicity, birthplace and language, for example. Including in our analysation of the politics of belonging, we extend the boundaries to include and move beyond identity, allowing us to more carefully pin down the tangible acts in which women engage to make community in spaces where socio-cultural identities matter but may not always be shared. In addition, we think more deeply about how women use the physical space in which they are situated to make community. We understand and integrate ‘community-building’ and ‘belonging’ in this context as closely integrated concepts. That is to say that in engineering ‘community-making,’ we understand that women are also co-producing belonging. Similarly, Block (32) emphasises this point in a two-part definition of belonging: (1). “First and foremost, to belong is to be related to and a part of something” and (2). “To belong to a community is to act as a creator and co-owner of that community.” In contrast to maternity care in a one-to-one format with a clinician where care is often not co-created and co-owned, group care offers an opportunity for social and relational elements which make up belonging to be observed.

‘A sense of belonging’ enhances social capital shown to improve individual and collective health and well-being. Public health data show a developed sense of belonging increases resilience (33), social participation (34), connectedness (35) and both community and self-engagement with public health messaging (36). A depreciation of belonging amongst group members in societies also negatively impacts health choices and thus their life chances. For example, a recent study illustrates how a lack of belonging was closely associated with vaccine hesitancy amongst socially excluded groups across several different countries (37). GC_1000 implements a group-based model of care, different from the traditional one-on-one consultations women typically receive all around the world. The key premise for providing services in a group include peer support, community building and sharing information and resources, making it a model of care to spotlight belonging.

Demonstrating improved neonatal health outcomes amongst groups typically underserved by maternity care such as adolescents and African American women (38), group care is increasingly being implemented in various settings across the world. We acknowledge that vulnerable conditions in which many women live make poorer maternal–infant health outcomes more likely and that experiences are influenced by a setting’s sociopolitical and historical landscape. “Vulnerability” is a contested and sometimes controversial term often stripping women of self-agency and resilience. Here we do not equate women living in vulnerable situations, who are often underserved, with being vulnerable women. Instead, we problematise the reach of maternity services which render communities ‘underserved.’ In this article, data from different settings describe maternity services and maternal–infant health initiatives that systematically fall short in serving women based on distinct and intersecting social positions such as: geographical location (i.e., rural settings), immigration status (i.e., migrant communities, refugees), public vs. private health care users and racial/ethnic minority groups, to name a few. In our analysis, we situate some women accessing group care as ‘underserved’ to describe more broadly how women in some situations lack access to the full extent of services which are of quality and acceptable to women who use them.

## Aim and research questions

GC_1000 aimed to develop evidence-based strategies and resources to support the implementation and scale-up of GANC and GPNC within different health care systems (18). Adaptations were applied at the country level to account for contextual differences (e.g., types of services, geography, population). The aims of the analysis reported here were to explore how and to what extent community-making engenders a sense of belonging amongst group participants and to understand how these experiences of care underpinned with ‘a sense of belonging’ may address the intersection of social well-being and health.

Within the context of the GC_1000 evaluation data on women’s experiences, we asked:

1. What are the ways that GANC and GPNC participants perform affective community-making?
2. How do these acts of affective community-making cultivate a sense of belonging within the group?
3. What inferences can be made from investigating a sense of belonging in the context of group care about how health care may operate as a social institution?

## Materials and methods

### Study population and sample

The GC_1000 project identified the study sites with attention to ensure diversity of participants across countries. For women participating in care, attention was placed to ensure diversity to include women from various socio-economic backgrounds, racial/ethnic groups as well as first-time and returning users of maternity care. In some settings, there was a specific focus on groups to serve refugee or migrant women (18). The overall sample size was determined by the available and relevant populations for each setting within the study period (between 2020 and 2023).

Purposive sampling was used to observe a selection of groups in each study site and country, in initial and later stages of the care process [more details can be found in McCourt et al. (13)]. In this paper we draw primarily on focus group discussions (FGD) and/or one-to-one in-depth interviews (IDI) with women which followed an observed sample of care sessions with those participating in group care (Table 1).

**Table 1:** Sites and local settings.

Country	Health care system	Main provider of antenatal care or postnatal care	Experience in group care	Population of women
Belgium	Midwives and GPs monitor pregnancies in primary care; however, most women are followed up by a gynaecologist in secondary care during pregnancy. There is a fee for service payment system, supported by social insurance.	ANC is provided mainly by obstetricians/gynaecologists.	Few group care services were in operation prior to the GC_1000 project. The project’s implementation sites did not have prior experience with GC before participating in the study.	Focused on women in vulnerable situations, the GC sessions were organised in community health centres, or in a hospital. Two of the three sites provide services to populations with mixed ethnic backgrounds and deprived socio-economic communities.
Ghana	Health sector is primarily funded by the National Health Insurance Scheme (NHIS). The ANC and PNC services are delivered by Primary Health Care financed through NHIS where pregnant women can access services at no cost.	ANC is provided by midwives, and also by auxiliary cadres where there are insufficient fully qualified midwives.	GC had initially been piloted in a different site and geographical location. Within the GC_1000 project, the site had no prior experience with the model.	A rural location where the communities faced low literacy levels and inadequate access to antenatal care.
Kosovo	ANC services are offered in both primary and secondary public healthcare institutions and private clinics, typically provided by healthcare professionals such as obstetricians/gynaecologists, midwives, and family medicine physicians, with the specific provider depending on the woman’s preferences, and ability to pay for private sector consultations. In primary healthcare centres, midwives usually conduct regular check-ups and refer patients to a gynaecologist if needed. Public institutions charge a small, symbolic fee for these services.	ANC is provided mainly by obstetricians/gynaecologists.	Before the initiation of GC_1000 project, there were no other initiatives related to GC in Kosovo.	Initially implemented in urban areas and sessions were attended by low-risk pregnant women.
The Netherlands	Social insurance-based free access to maternity care; Low-risk ANC is based in primary care. Hospital birth recommended only for those with high risk factors.	Midwife-led for all low-risk pregnancies; Independent midwives provide care in community for low-risk or clinical midwives in hospital for higher-risk women.	GC has been piloted in different community paediatric services for children and families in general and children and families in asylum-seeking centres specifically. Ten years of experience with GC prior to GC_1000 with its own GC institute, CenteringZorg. About 30% of midwifery practices offer GC though not implemented routinely. Postnatal GC is on the rise, but not yet widely practiced.	GC was implemented in four sites where it was not previously practised with a specific focus on neighbourhoods with more diverse populations; it was also implemented in three centres where women participating were seeking asylum and were from different countries: e.g., Nigeria, Syria, Sudan, Yemen, Ukraine and Eritrea.
South Africa	Inequities exist between the public and private health care sectors. The public sector serves 80% of the population who can access maternity care at no cost to the service user, however there are variations in quality of services across the system.	Typically, in the public health sector, women are seen by midwives or nurses. However, the GC_1000 site location provided services to women with both low- and high-risk pregnancies.	This was the first time group-based antenatal care was integrated in the public health system.	Urban, low-risk women in the antenatal clinic of a referral-level hospital that also caters for women at higher obstetric risk.
Suriname	Pregnant women receive ANC in public primary care facilities and private general practitioner (GP) clinics and are referred to secondary care in the third trimester or earlier if needed.	ANC is mainly provided by obstetricians/gynaecologists during the third trimester and in the first trimester by midwives/GPs (primary care), and in the remote interior by skilled health assistants.	GANC was developed and implemented in 2014 in three hospitals in the capital city as a pilot project. In 2019 it was introduced in primary care but not continued due to the COVID-19 pandemic. GANC and GPNC were implemented as part of the GC_1000 project in primary health care facilities and a hospital setting.	Urban cities: the capital Paramaribo and district Wanica. When participation is self-selected more highly educated women participated. Implementation focused on inclusion of partners in all sessions.
England, UK	Most women access maternity services via England’s National Health Service (NHS), a publicly funded healthcare system.	Midwives in the UK provide most antenatal, intrapartum and early postnatal care and are lead professionals in care for women with low-risk pregnancies. Midwives refer women to obstetricians as needed based on risk assessment.	A pilot project was conducted in one service from 2008 to 2010. The REACH Pregnancy programme conducted feasibility studies and a multicentre RCT of Pregnancy Circles Trial commenced in 2018. The GC_1000 project built upon two of the trial sites, where the model had already been piloted (13).	South of England, one site with previous experience of group care extended the model to include postnatal group care, called Pregnancy and Parenting Circles; the other site integrated GANC with caseloading midwifery teams, which provided continuity throughout maternity care. All women, regardless of parity or pregnancy risks were invited to join in the sessions.

### Data collection procedures

Mixed methods of data collection including observations of training and group care sessions, survey of participants, facilitator record forms and interviews or focus groups with stakeholders, facilitators and group care participants were used to obtain a rounded picture of the experience of implementation and of group care, from service, professional and user perspectives.

Researchers conducted interviews in the local or preferred language of the research participant including: Dutch, French, English, Albanian and Kusaal as, in some contexts, group sessions were multilingual. In Belgium, some research participants were able to more comfortably communicate in English compared to Dutch. Therefore, interviews were carried out in English with these participants. And in the UK, two focus group interviews required foreign language and British Sign Language interpreters. Interviews not conducted in English were translated from the original language to English using different methods. For example, in Ghana and Kosovo, interviews were conducted in the local language and transcribed by a translator with expertise in the local language. Other interviews in Suriname, The Netherlands, Kosovo and Belgium where transcriptions in English were not already provided, automated translation services were used to translate into English for the analysis and reviewed for accuracy by original language speaking researchers. Quotes of significance to the analysis were cross-checked with co-authors with both contextual and linguistic expertise.

### Data management and analysis

To address our aims and research questions, we adapted a meta-theme analysis using an abductive approach to explore women’s experiences of group care with a focus on identifying ‘affective community-building’ as a catalyst for creating ‘a sense of belonging’. Following Wutich et al. (39)’s approach as a guide to meta-theme analyses for cross-cultural research, we focused on country level qualitative data on women’s experiences of group care. Focus groups and one-to-one interviews included in the analysis were conducted by each country’s local researchers. Transcripts for each country were analysed as a part before being collated into a whole. Transcripts in each country were coded using a framework that was developed using an initial sample of interviews conducted across all countries. However, the abductive approach to the analysis allowed enough flexibility to identify context specific themes and amend the framework as needed. Codes found from each country were then compared to identify and build upon cross-country themes.

The table below highlights the definitions of key themes identified through the analysis (Table 2).

**Table 2:** Key theme definitions.

Key theme	Definition
1. Socio-spatial building	Encapsulates the interactions between people, societies and the physical environment which they occupy, emphasising how they shape one another (41). We apply this concept to group care to understand how the physical environment within the group session as well as the people who make up the group interact with and shape one another.
1.1. Collective Agreements	Verbal or written confidentially agreements and other ‘ground rules’ discussed within the group.
1.2. Boundary setting	Setting boundaries around the physical space.
1.3. Care Gestures	Formal socio-spatial building such as building confidentiality and boundary setting, help to feed into to ‘care gestures’ amongst group participants. In affective community making, women engage in gestures of care toward one another.
2. Communal building of health literacy	Captures how women utilise lessons of health and well-being from sessions and translate that knowledge to suit needs which exist beyond the boundaries of the inner community of group care. It is defined here as an omnidirectional knowledge transferal applied beyond the perinatal period and inclusive of community outside group care.
2.1. Individual Health	During the self-checks women learn about their own health and well-being while doing the assessments and through discussion in the group. This exchange is the starting point for communal health literacy.
2.2. Community Health	Distribution of skills to collect and interpret personal health information positioned women as collaborators of care. The group care format allows for more information to be discussed and reflected upon, such as common health care measurements like blood pressure readings.
2.3. Partner Involvement	Inclusion of male partners extended communal health literacy, enabling male partners to be more informed as supporters in pregnancy, birth and parenthood.
2.4. Social Care	Women defined how group care sessions in this context formed a part of their social care and reiterated how this strand of affective community making could be further embedded in the model to improve the experiences of women and their children.

## Results

Women’s experiences of group care across different settings illustrate that the ways in which women participate in community-making to engender a sense of belonging in the context of group care are complex and dynamic. Clear examples illustrate how women’s active participation cultivated a sense of belonging, with the potential to mobilise community action. First, two major themes outline how women utilise social interaction and their physical environment to engage in community-making, called (1) socio-spatial building and then further elaborate on how the investment the GC model places on demystifying health literacy via self-checks elevates the wider community via (2) communal building of health literacy. And finally, we further unpick the complexities of (3) cultivating a sense of belonging by exploring the tensions and opportunities with a focus on migrant women, language and inclusion (Table 3).

**Table 3:** Interview type and number of participants.

Country	Interview type	No of participants
UK	Site 1:3 FGDs & 5 interviews (*n* = 15); Site 2: 4 FGDs & 6 interviews (25).	40
South Africa	5 FGDs +2 individual interviews	26
Belgium	6 in-depth interviews	6
The Netherlands	5 antenatal & 9 postnatal interviews in-depth interviews	13
Suriname	2 FGDs with 7 antenatal group participants, purposively sampled across groups	13
Ghana	3 FGDs	21
Kosovo	4 FGDs	17

### Socio-spatial building

One way in which women affectively create community within the model is via socio-spatial building. The interaction between sociability and physical space is in part structured through group care’s multiple strands of continuity including site location, group participants and facilitators. Socio-spatial building is a layered process within the group, constructed in part through the physical proximity of women with a shared identity and somatic experience of pregnancy and mothering; and in many instances, returning to the same space and health practitioners for their care. The nuance of socio-spatial building involves not just the physical space but also an extension and contraction of space between the group and community. For example, in South Africa, the women in one focus group illustrate how acts of socio-spatial building on behalf of group care members, and particularly the pregnant women, are purposeful, and distinct from the existing environment constituting maternity care. In other words, women describe the ability to speak openly about their experiences and in turn learn from one another, not solely because women are brought together, but because measures of community-making are activated to create a space of open communication through their socio-spatial building. In part, this is because group care facilitators are trained in the practice of group facilitation. Community making depends on these procedural factors which derive from intentional planning and training that occur months prior to implementation.

*South Africa, FGD, 15th of February, 2024:*

*SA Woman 2: The environment can be fine, but the people in your group make things even better… Because, like I say…if the people judge you, you feel uncomfortable….*

*SA Woman 3: You’re not going to open-up.*

*SA Woman 2: … whether it’s your second or third child, and you do not know, we were all comfortable with one another to ask. There’s certain stuff that I learned from another mom, and there’s stuff maybe that somebody else learned from me that they [did not] know about.*

*Researcher: Yes.*

*SA Woman 3: Even the facilitator made you feel welcome and open to ask anything…You could ask anything from [the facilitator].*

The active and intentional acts of socio-spatial building may contribute to women’s positive experiences of the model. Many women who participated in the study described feeling comfortable with one another, as reflected in a focus group in Kosovo.

*Kosovo, FGD, 24th of May 2023:*

*KSV Woman 1: … there were 7 or 8 other participants in the same room, it did not matter because we felt comfortable with each other.*

Women’s socio-spatial building is contextualised within the model’s facilitative and interactive learning approach. Together, with a foundation of shared physical space and common experience (i.e., pregnancy, parenthood), some women in this model demonstrate the ability to engage in deliberate acts of socio-spatial building constituted by: (1) collective agreements, (2) boundary setting, (3) care gestures to build-up conditions where open communication, trust and emotional vulnerability have opportunities to emerge within the group care context, forging a sense of belonging to the group.

### Collective agreements

In some settings, such as England, confidentially and other ‘ground rules’ were discussed within the group and agreed verbally in the first session. In South Africa, by contrast, confidentially agreements were made via voluntary signed agreements:

*South Africa, FGD, 20th of September 2024:*

*SA Woman 4: Women love to talk. It does not take long…*

*SA Woman 3: Yeah. It does not take long. One person will start, and the rest will follow…*

*SA Woman 2: And the confidential thing.*

*Researcher: The confidentiality.*

*SA Woman 2: That matters. Because [it] is why we feel so comfortable with each other because we know…*

*SA Woman 1: [intervenes] …it stays in the group.*

*SA Woman 2: Yeah. None of us [are] going to go and blurt out to someone else, because we signed a confidentiality form. We know for a fact, because our signatures are there, that what happens here, stays here.*

The data show how collective agreements are mutually assembled ‘codes of privacy’ established via confidentiality forms and ground rules. In some instances, group facilitators helped to reiterate the group’s code of privacy.

### Boundary setting

Some women described the importance of setting boundaries around the physical space to only include group care participants as an important way to protect the privacy of participants. In South Africa, for example, one woman raised how the disruption to a private physical space can detract from women feeling comfortable to always speak freely.

*South Africa, IDI, 1st of June 2023:*

*Researcher: What could be changed or improved about group care?*

*SA Woman 2: I think it’s just the place where it was held…people were walking up and down [the corridor]… If they could be like in some separate place for them, because some people could hear… if we [spoke]… I do not mind but for some other person [in group care] like their [privacy].*

To contrast, in the Netherlands, women in a focus group discussed how a private physical space contributed to the group’s dynamics. Their statements illuminate how setting boundaries to protect who can enter the physical space is an active approach to how women maintain affective community building.

*The Netherlands, FGD, Date of FGD unknown:*

*NL Woman 1: My favourite thing [about the group] is the privacy. When we are in the room, no one comes in that we do not know. I like that, a lot.*

*NL Woman 2: Because I do not discuss [our conversations] with other people. So, since we are in the group I know it’s not going to go out, so I know to discuss it there. I love it.*

### Care gestures

In England, an example of this is raised in a focus group. Women described not only having an awareness and concern for their own health and wellbeing, but also extended this to their other group members. Having learned how to take their own blood pressure, in certain instances where a woman would come into the group session distressed or unsettled, other women in the group advised her to wait to take the blood pressure reading so that the results would not be skewed. In addition to this, on at least one occasion, group participants would offer the woman a cup of tea and a biscuit as a care gesture, waiting for her to be relaxed to take her blood pressure reading.

*England, FGD, 25th of April 2023:*

*ENG Woman 1: …we’d all be like, ‘Right, you are out of breath - wait a minute. You wait a minute [before taking your blood pressure] …’*

*ENG Woman 2: If one of us were on the phone still dealing with something, we’d be like just [tell them to take their blood pressure reading] later…have a tea and a biscuit.*

*Researcher: So, you helped each other?*

*ENG Woman 1: Yes. Yes, we knew if someone’s blood pressure was a bit high, we’d say, ‘You’ve just walked. You wait there a minute.’*

Similarly in Kosovo and South Africa, women elaborate on how these care gestures also take the form of practical and emotional support as well as camaraderie.

*Kosovo, FGD, 9th of June, 2023:*

*KSV Woman 4: We are like friends, for everything we need. When we have problems or we are happy, we always talk with each other. We also sent pictures to one another, pictures of the babies or pictures of us with the babies, or some memes related to parenthood.*

In South Africa women expressed how they used WhatsApp beyond the group care sessions to share their experiences. For example, when unable to sleep in the middle of night, having someone to speak to was appreciated.

### Communal building of health literacy

Women provide examples of how affective community making is applied to: (1) Individual Health, (2) Community Health, (3) Partner Involvement, (4) Social Care, and (5) Including Wider Community in Group Care which develops communal health literacy.

### Individual health

In group care, women are taught to carry out self-assessments of health measures. During the antenatal period this can consist of blood pressure readings, weight measurements and signs of common pregnancy complications (e.g., preeclampsia, gestational diabetes), depending on the setting. In some contexts, women carried out the blood pressure assessment on themselves and in others, this was something that women performed on each other (i.e., checking another woman’s blood pressure). Group facilitators, usually health care workers themselves, share skills with group care participants on how to measure their own blood pressure readings and interpret the results. Women illustrate a profound shift of power through this knowledge exchange and how it provides additional skills in monitoring their health.

*South Africa, FGD, 15th of February 2024:*

*SA Woman 2: It made me more knowledgeable about healthcare. Sometimes, they take your blood and…we do not know what is the readings. In group care, we learned when your blood pressure is too high, when it’s too low. Your urine, we learned that as well. So, that was helpful for me.*

*Suriname, FGD, 6th of March 2021:*

*SR Woman 1: I learned a lot during the sessions and, of course, I took it seriously. I also always liked the fact that we were allowed to measure our blood pressure ourselves and stand on the scales [to weigh ourselves]…Those were all very nice experiences. It’s the little things that made me happy.*

In Belgium, women ascribe a sense of empowerment to the inclusion of self-checks, showcasing how it is important for women to actively be involved in their care.

*Belgium, IDI, Date of IDI unknown:*

*BE Woman 3: [It’s positive] that we have a say in our own [care], for example, measuring blood pressure… I have the feeling that in these [group care] sessions that you have the ropes in your hand, and that you can give more direction. [To say], ‘I want to know that or I do not want to know that. Or I want to ask this question.’… Should the doctor or midwife be in charge? I do not like that as much. I want more myself [to be in charge]… Before my appointment I have an idea of what I want to ask, but instead I spend the time answering the doctor’s or midwife’s questions and I forget my own.*

Women in focus groups in England described how health assessments learned during group care sessions in pregnancy were also transferred to their knowledge and understanding of their health and well-being outside of the group care sessions.

*England, FGD, 4th of July 2023:*

*ENG Women 3: When I went for my check with the general practitioner, I also had [my blood pressure checked]…I felt like I knew more about my blood pressure. It wasn’t just someone taking a random measurement and putting it on a chart. I could understand where my blood pressure was and understand if my ankles were swollen. [I understood] what was going on in my body. That made me feel a bit more connected to my body when I was pregnant and I kind of wanted to know how things were afterwards.*

*ENG Woman 1: Yeah, I had my blood pressure taken with the GP yesterday and when she read the numbers back to me, I know what it meant.*

### Community health

Women across different countries described how access to this knowledge empowers its use to aid and support members of community outside of group care, including family members.

In South Africa, a woman in a focus group describes how the information she learned via self-checks positions her as holder of knowledge.

*South Africa, FGD, 15th of February 2024:*

*SA Woman 3: [The health checks are] something we can do at home with somebody else that’s maybe feeling a little bit ill. Now you know how to do a blood pressure test.*

*Researcher: So, it’s a skill now that you have?*

*SA Woman 2: Yes.*

Again, in South Africa, a woman explains how knowledge gained from performing group care self-checks and other health assessments would also extend to caring for her baby.

*South Africa, FGD, 19th of July 2023:*

*SA Woman 2: It will be so cool that I’m going be a mommy now. [It] is going to be good for me to know how to check [their health] …I really did not expect to learn what I have been taught here, I’m very glad that I agreed [to take part].*

In focus groups in Suriname, one woman describes how self-assessment practices reflected in the group care self-checks are a valued skill to have and that this provided an opportunity to learn how to carry out these skills which she had otherwise missed. She goes on to describe how learning to measure and interpret blood pressure readings would be helpful not only for herself but for elder members of her family.

*Suriname, FGD, 1st of April 2023:*

*SR Woman 1: I have always been taught at home that you should be able to measure your blood pressure yourself and of your father, your mother, your grandfather, your grandmother. So, I’ve always seen that everyone should be able to measure their blood pressure themselves. But despite this, I never did it myself. I did not know it and I was never in a position to do so…During the group care sessions I recently measured [blood pressure] for the first time for someone. I think in the long run, this is good. Not only for yourself, but also for other people at home. If you are [suddenly] in front of your father or mother and they feel unwell, first thing you are going to do is measure their blood pressure. So not that you only do it for yourself, but also for the people you are with [and who are] around you.*

Women candidly described the group sessions as a centre for information exchange. A place where they can re-use helpful resources to inform community members without access to the group space. One example from South Africa illustrates how it supports women as pillars of their families and communities.

*South Africa, FGD, 18th of April, 2023:*

*SA Woman 1: Both of my cousins are also expecting. So, we are basically giving birth the same month. They are not part of group care [nor do they have] somewhere they can go to exchange information… I feel like I help them in a way, so that they are not alone given that we are expecting babies together. We have that kind of information sharing arrangement.*

*Researcher: [Are you] becoming like a leader?*

*SA Woman 1: Yes.*

### Partner involvement

The inclusion or desire to include partners, particularly male partners, varied as some women felt that group care session most often flourished as a ‘women only’ space. A few women described that the presence of male partners might inhibit their ability to speak freely in sessions and that it offered women an opportunity for independence. However, the focus here highlights that when women did discuss the inclusion of partners (i.e., fathers), this built upon their acts of affective community making.

Group care sessions in Ghana were situated in a rural setting where access to general healthcare had been limited. A woman in a focus group described how inclusion of men in group care sessions may help to alleviate suspicion about new information learned in the sessions.

*Ghana, FGD, April 2023:*

*GHAN Woman 4: There are still problems in our communities because most of our husbands still do not believe whatever we come home to tell them about [group care] trainings, therefore if they can be in the group meetings it will help resolve some of these problems.*

In South Africa, women described examples of how they would relay information learned in the group session to their partners resulting in more informed and empathetic supporters.

*South Africa, FGD, 19th of July 2023:*

*SA Woman 4: I would tell him what we discussed [in group care]. He [would] be more interested because it’s so new and good for the both of us. I think it helps because he now knows what I’m going through and he is more compassionate.*

Focus groups in Suriname reveal how the inclusion of partners in the group sessions allow for women to connect more deeply with their partners as a couple and in their responsibilities as parents which is unlike other health care settings they had experienced.

*Suriname, FGD, 1st of April 2023:*

*SR Woman 1: What I really like is that the men also have a place. You do not really see that anywhere. The men also have a place to let out [in the group care sessions]…because suppose your wife is afraid or does not like something, then you have the knowledge…*

*SR Woman 2: Yes, and [the couple] can bond… in pregnancy there can be times when the partners do not understand each other. And then suddenly you find yourself in a group of people, where the opposite sexes understand each other… So, then you have some kind of support.*

*SR Woman 2: Everyone supports each other. The women support each other, the men support each other too.*

### Social care

Framing health care as a social institution inspired the consideration of the ways in which group care may touch upon the women’s lifeworlds such as their socioeconomic and mental health needs. Some of the women who participated in postnatal group care in the Netherlands were refugees and asylum seekers, people who often face challenges of recovering from particular types of past trauma, adjusting to a new country and awaiting settled status in addition to the stresses that often come with caring for their babies and perhaps other young children. Although, social care was not a way in which women sought to engage in ‘affective community making’ across the different countries, it was an important finding in data from The Netherlands worth highlighting.

*The Netherlands, IDI, Date of IDI unknown:*

*NE Woman 8: For me personally the most important sessions where about how to take care of yourself as a mom, how to handle stress, because it can be overwhelming. It is easy to fall into depression, so the different things that we talked about during that session where really helpful. I remember I was at the verge of going into depression because it was too much for me to handle. What we talked about then really helped me. They talked about how you can take care of yourself: you can go for a walk, you can pause, you do not have to do everything. You have to also really bring your health into account. I still go with that. If I need to pause, I pause, if I need rest, I rest, If need to do what I like, I do it, not everything is about the baby, it is also about me. That really helped me.*

In this in-depth, on-to-one interview, this same woman goes on to describe how social care needs could be further embedded in the group care sessions. Having previously mentioned how the features of the model where a vehicle to learn important self-care techniques, she illustrates how the model could apply those same mechanisms to sharing social care information and resources.

*Researcher: Is there anything about how you are doing or about the group session that we have not talked about that you would like to talk about?*

*NE Woman 8: I think we need to talk about work. Because sometimes I feel like maybe I need to find work, I need to get a job. [I need to learn] where can I leave the baby, how can I handle it. That’s something that I wonder about. [I wonder] if there is any facility or daycare where [the baby] can play with other kids…But I do not know if there is that. I think that would be really of help with the parents.*

### Informing wider community about group care

Many women who participated in GANC and GPNC described the unique position of the group care model, and some even highlighted how this compared to conventional one-to-one maternity care. Although health care systems and care delivery vary by setting, a central point that women made as a benefit of group care was the community building elements the model provided. None more thoughtfully explained like a woman in Suriname.

*Suriname, FGD, 1st of April 2024:*

*SR Woman 1: When we talk about what is important about care during pregnancy. Then I would say, especially with the first pregnancy, it’s everything you do not know. Pregnancy is new, having a child is new. Nine months of pregnancy and certainly childbirth, I think, are the most vulnerable times in a woman’s life. The bottom line is that it puts a lot of stress on you as a woman and also on your relationship [with your partner]. And what’s important is everything you did not know about pregnancy, hormones, child development, and what to expect from healthcare…when I talk about healthcare, the difference between individual care and group care. I feel…when you go to one-on-one care, you feel like [the health care professionals] do not have the time…You almost do not have time to think about a question because you think ‘oh I have to leave because there are others waiting’…It’s only when you get to the group session that you [realise] what you do not know. So, that’s the beauty of group care compared to one-on-one care.*

Many examples emerged in the analysis of how women wanted to share this type of care with other women in their lives and in their communities. Some of the women offered ideas in how to improve recruitment to the model and advertise the model with other expecting parents.

In South Africa, women reflected on their experience of the group model of antenatal care and how it would have been useful for other women in their lives.

*South Africa, FGD, 18th of April, 2023:*

*SA Woman 3: Even my mom is so surprised about how much knowledge that I have gained [from] this group…[She said that she wished she] had that back in [her] days. So, yes, it is helpful a lot.*

In this same focus group, another woman describes ways in which the model could be upscaled in South Africa to reach under-resourced communities.

*SA Woman 2: If the [group care] programme is successful [in the GC_1000 project then] it should go to rural pregnancy clinics. Because I think, the patients there definitely need it…Because in some situations…young girls get pregnant, give birth and then their grandparents must take responsibility to raise the baby because the parents do not have the knowledge to do it. So, [if young mothers were able to] receive all the information that we have got so far, I think, maybe it will change their minds.*

Focus groups in Kosovo also reflected a similar sentiment that the model should be more widely offered across the country. It reflects how women feel that this model of care serves an unmet need for many women.

*Kosovo, FGD, 16th of June 2022:*

*KSV Woman 2: If this circle had been more distributed or if it had been in every city of Kosovo, or even outside of Kosovo, it would have been very good to distribute all this information. And I feel sorry for those who have gone without experiencing these information and experiences.*

Similarly, in England, women identified gaps in the care of other women in their lives and emphasised how their experiences might had been improved had there been an opportunity to take part in group care.

*England, FGD, 25th of April 2023:*

*ENG Woman 2: When my sister-in-law had my nephew, she did not get any of this. She had not been offered [group care]. So, when I was telling her about [what we learned], she said that [in one-to-one antenatal care] she only got that information if [she] remembered to ask for it.*

Focus groups in the Netherlands also reiterated how women who experienced group care felt that other women in their community could benefit from this model of care.

*The Netherlands, FGD, Date of FGD unknown:*

*NE Woman 1: My neighbour also had a son also, the same age as my baby. I think she might like it if [the group care team] sent her a letter [to invite her to join].*

*NE Woman 2: Yeah, maybe [recommend group care to] people in the [asylum seeking] camp…people should come to this place. It is good way to learn a lot of things about how you [raise] your kids.*

### Cultivating a sense of belonging: tensions and opportunities

#### Migrant women, language and inclusion

Health care services are spaces in which people in society come together. Bringing a group of women together across sociocultural and linguistic backgrounds can sometimes present delicate tensions as well as opportunities for creating a sense of belonging. Developing ‘a sense of belonging’ in the context of group care is textured and multi-dimensional, illustrating the intersectionality of identities within expecting or new parents. Some aspects of identity or social position of an individual may be represented and serviced in the group context, while other aspects of their identities or experiences may feel underrepresented or isolated. This could lead to feeling partially excluded from the group, unable to fully express their concerns.

In this analysis, women with backgrounds furthest from the dominant group in their country’s context (e.g., pregnant women or new mothers settling into a new society as an immigrant, refugee or simply a newcomer to the area) provided the most visible examples of possible experiences of belonging. For example, many of the group care cohort sessions involved in GC_1000 were carried out in the local language. However, in more ethnically diverse and multicultural areas, multilingual groups were commonplace. In some contexts, the sessions were conducted in English, the most commonly held language, to bridge communication between speakers of different languages. For instance, in Belgium, a few of the group care cohorts included women from other countries who did not speak Dutch or French fluently, and therefore sessions were mainly conducted in English. On the other hand, in England where British English is the dominant language, interpreters were active participants in the sessions to support women who communicated in other languages.

Particularly in interviews and focus groups with these women, the intricacies of developing a sense of belonging were exposed – not only in the antenatal or postnatal group itself but also through interacting with other women in group care. Those new to the society were able to find additional support in getting settled in their new environment. For example, in England, a woman agreed via an interpreter that she found group care assisted her adjustment to her new country.

*England, FGD, 12th of June 2023:*

*Researcher: I’m just curious about how the group sessions helped people to adjust to a new place. Has it helped at all?*

*[Interpreter speaks to ENG Woman 3 translating from English to the woman’s preferred language. ENG Woman 3 and the interpreter talk to each other in the language spoken most comfortably by the group care participant].*

*Interpreter: I was just asking [ENG Woman 3], ‘Do you think it was a good introduction to the culture?’ And she was saying, ‘Yes’.*

Even women who are native to the country, but new to their local area, described group care as a vehicle to help in adjusting to a new place.

*England, FGD, 25th of April, 2023:*

*ENG Woman 5: For me, I found that because I’m not from the area, it would be really nice to actually meet people in the area… again people that were going through the same thing. Straight away I thought what a fantastic idea. It really was. It’s worked well. When I have the next baby, I would opt in [to group care] again*.

In the Netherlands, in the asylum-seeking centres, group care was centred on the postnatal period, particularly serving women and their children who were seeking asylum from countries such as Yemen, Nigeria, Syria and Sudan. One woman described how involvement with other participants in group care provided an opportunity to not only be exposed to new parenting practices, but to also share her own.

*The Netherlands, IDI, Date of IDI unknown:*

*NL Woman 3: We talk about the children, the rules in the life, in the house…Maybe another mother likes this rule, and she’ll make it her own.*

*Researcher: You learn from each other.*

*NL Woman 3: Yes. I need advice sometimes and when we go [to group care] I am happy, because I learn something I did not know before.*

The idea of “rules in the house,” as described by NL Woman 3, illustrates that even though women come from different places, there are some common ideas about parenting that transcend countries and backgrounds. Group care provides a space to find and share these common ideas about parenting.

In Belgium, there was no official translator in the group. The group care facilitators spoke Dutch, English and French. If there was a native speaker of a language not commonly spoken amongst the group, facilitators tried to ensure that at least one other person in the group spoke the language. Women navigated multi-lingual dialogue within group sessions and in some instances worked with each other to communicate across linguistic barriers. One woman, a non-native speaker of Dutch or French, described the ways in which women reached beyond the challenges to having conversations in a multi-language context:

*Belgium, IDI, 12th of May, 2023:*

*BE Woman 1: We shared and then they translated. One [woman] translated maybe in French, the other in Dutch so that we could all understand. We also used cards with pictures. There was a lot of nonverbal expressions that we shared. So, even though the language can be sometimes challenging, I felt there were real moments of connection even when we did not know what the other was saying. We could see in their faces or body expression, how they felt.*

This example from the Belgian context of group care illustrates how women not only work together to create lines of communication, but that they also appreciate the efforts to do so, even when it does not always work. Though this analysis focuses on the process of community making, a spotlight on how some women experience and benefit from ‘a sense of belonging’ to the group, highlights how the model works at the intersections of health and social care.

## Discussion

Community building is a complex process where power dynamics of the wider society are often at play. From this analysis, we find that the facilitative and group-based elements of group care sometimes provide opportunities for group participants to transform those power dynamics. Much of the literature on group care describes women’s satisfaction of the model (26) and that within their cohorts the ability to ‘speak openly’ is space where learning occurs (17). This analysis takes this argument a step forward, highlighting that to arrive to a place of open communication, women actively engage in community making to achieve a sense of belonging to the group. We found that women engage in affective community-making in three key ways via socio-spatial building: (1) collective agreements and exchanges via confidentiality contracts, (2) boundary setting, and (3) care gestures. Through acts of community-making, women build up ‘a sense of belonging’ to their peers and facilitators in group care. Much like Marco Antonsich’s framework on belonging, we find similar complexities in the politics of how women connect with each other through and beyond shared identities, stages of life and their physical environment.

In line with public health literature which highlights the benefits of a sense of belonging, we find that where women locate a sense of belonging through their experiences of group care, they are then mobilised to bring knowledge and resources back into their families and the wider community. Evidence on community mobilisation derived from women’s participatory groups identify it as a strength of group-based models, but few articulate the route to community mobilisation. Mehay et al.’s (30) realist review of literature about group antenatal care identified that social support and community building are considered key mechanisms through which group care ‘works’ for people, across different contexts but that there is only limited elaboration of how community-building works in practice.

Data from this cross-cultural meta-theme analysis, more finely illustrate that when women find beneficial resources to holistically nurture themselves and their children, many of them find ways to share and advocate for similar resources to others exemplified via the pursuit of communal building of health literacy: (1) Individual Health, (2) Community Health, (3) Partner Involvement, (4) Social Care, and (5) Informing Wider Community about Group Care, and about caring for one’s health. For many women in the study, it started with health self-checks such as blood pressure measurements as a part of implementing the group care model. In learning how to look after themselves, women described how the information could also be utilised to better their families and communities. The knowledge that women gained, both from group facilitators and participants, was identified amongst women to share, particularly with their male partners. A few women commented on how in sharing this information with their partners, they may find improvements in not only the support they received but also in their relationship. Even though the inclusion of partners in the group care is mixed both in the literature and in this research study, some women stated that some involvement of particularly male partners might also offer a space for them to share their experiences of fatherhood.

The motivation to improve health, wellbeing and life chances extended beyond the participants’ immediate families and networks. As Gilson goes on to describe health care as a social institution, “Although patients may be primarily concerned with getting well by getting good health care for themselves, citizens may be equally or more interested in the role of health systems in allowing the attainment of other goals.” Women in this study identified ways in which the model could be further utilised to improve society. For example, in the case of women seeking asylum in the Netherlands, one woman stated that the group care sessions could discuss other social determinants of health such as employment and childcare. Other women across different settings, such as South Africa, Kosovo, England and the Netherlands, shared how the group care model could be upscaled to improve the lives from different communities and across the country. Group care research makes some connections to the cultural and social relevance of the model, highlighting how communities are mobilised through support and networking, “…group antenatal care enabled access to a community, especially those women new to the area…” (28). Results of this analysis provide a novel addition to the literature which indicates the stimulation of community mobilisation through group-based maternal health and social care, illustrating the role community-making and links to the actions women from knowledge, skills and resources developed through the model.

### Strengths and limitations

Data collected in the GC_1000 project were formulated as a part of an implementation study of the group care model where the focus was broader than the subjects of ‘belonging’ and ‘community-making’. The interview schedule for group care participants was semi-structured, allowing for some flexibility to incorporate context specific research questions across each country involved in the project. The semi-structured interview format also allowed for open-ended responses to illuminate patterns within the data that would otherwise might have been unarticulated in structured interviews (40) which captured women’s experiences of belonging and how they went about creating community within the group context. Some research teams conducted more in-depth interviews whilst others collected more limited data from women involved in the study. Therefore, the meta-theme process of analysing data as described by Wutich et al. (39) was adapted to accommodate unequal levels of data. The interviews and focus groups drawn on here were complemented by research observations of the group care process and analysis of facilitators’ experiences and perceptions. While meta-theme analysis across diverse contexts had some challenges (i.e., unequal data amounts from different countries), it also provided the benefit of the ability to observe and analyse threads of shared and different experiences across diverse settings and maternity care systems. A further limitation was that the timeline for the study did not allow longer-term follow-up with participants to explore more fully ways in which community building might extend beyond maternity care itself.

### Concluding points

Social complexities are ever-present and reproduced within health care institutions. This study builds upon the evidence which highlights the strengths of group care, as a relational healthcare model, to address the holistic needs of women often underserved in conventional maternity services. In a cross-cultural meta-theme analysis of women’s experiences, we have identified the ways in which women actively take part in building a sense of belonging to their group care cohort. Our findings also contribute to wider research on women’s participatory groups which describe community mobilisation as a byproduct of facilitative, group-based models, grounded in liberatory rather than didactic pedagogy and using a strength- rather than deficit-based approach to healthcare. While previous studies have highlighted satisfaction and engagement with group care, findings from this study illustrate more finely the routes from women’s groups to community building and mobilisation. More research is needed to better understand how the claims of community-building in group care may be supported by the cultivation of belonging in this care approach and further develop the ways in which it may lead to better health for the wider community.

## Data Availability

The datasets presented in this article are not readily available because it is qualitative data. Requests to access the datasets should be directed to info@groupcare1000.com.
